# Supplementary material for: The gut microbiome is required for full protection against acute arsenic toxicity in mouse models
Source: Nat Commun. 2018 Dec 21;9:5424. doi: 10.1038/s41467-018-07803-9 (PMC6303300; doi:10.1038/s41467-018-07803-9)
Supplement: Supplementary file 1 — Supplementary Information [file 41467_2018_7803_MOESM1_ESM.pdf]

Supplementary Information

The gut microbiome is required for full protection against acute arsenic toxicity in mouse models

Coryell et al.

Supplementary Note 1. Arsenic dosing.

Selection of the dosing levels in this study (10, 25, and 100 ppm) was based on three criteria. First, for reasons highlighted below, acute arsenic toxicity studies in mice typically use high doses of inorganic arsenate (iAs^{V}) or arsenite (iAs^{III}), even though most humans are naturally and chronically exposed to much lower levels. The range of iAs^{V} dosing considered here was intentionally consistent with other studies using C57BL/6 WT and As3mt-KO mice (see Table 1 below). We also draw attention to the fact that a similar range of dosing in mice (1-50 ppm) was used to develop a ***human*** pharmacokinetic (PK) and pharmacodynamic modeling framework at the 2007 Annual Meeting of the Society of Toxicology[1]. Consequently, in terms of dosing *per se*, the levels we used are superimposable to those historically used.

Second, if the focus of a study is on toxicity, the level of exposure should be above the lowest-observed-adverse-effect-level (LOAEL) and somewhat lower than the immediate lethal dose (LD_{50}). Unfortunately, there is considerable variability in LOAEL and LD_{50} estimates for humans, but according to the ASTDR and EPA's toxicological profile on arsenic[2], the LOAEL for acute, oral exposure (Appendix A in profile) is approximately 0.05 mg iAs per kg body weight per day. Assuming (as recommended by ASTDR[2]) a 55 kg person drinking 4.5 L of water per day and a 0.002 mg iAs per kg per day daily food intake, this level of exposure equates to 611 ppb arsenic in drinking water. Also according to the ASTDR, death in oral arsenic exposures in drinking water >60,000 ppb can result in death. Thus, exposures between 0.6 - 60 ppm should be appropriate for studying acute toxicity.

Third, an allometric conversion must be done to normalize dosages between humans and animals. For example, the FDA suggests[3] that a human equivalent dose (HED) in drug exposure studies is:

$$\text{HED}\left(\frac{\text{mg}}{\text{kg}}\right) = \text{Animal dose}\left(\frac{\text{mg}}{\text{kg}}\right)\text{multiplied by }\frac{Animal\ Km}{Human\ Km}$$

where *Km* values are the ratio of body weight to surface area (mouse Km = 3, human Km = 37)[4]. Using this equation, 10, 25, and 100 ppm exposures in mice correspond to HEDs of 811, 2,027, and 8,108 ppb in humans. All three of these exposures are well within the ASTDR toxicity range described above. In addition, the first level (811 ppb) is actually lower than that of drinking water recently reported for an arsenic rich part of Chile[5]. The middle and upper exposure levels represent doses where we expected to see increasing (dose-dependent) levels of toxicity.

Supplementary Table 1. Range of doses in murine arsenic exposure studies.

Arsenical	Mouse (C57BL/6 = WT)	Dose (ppm)[a]	Reference
iAs^{V}	A/J	1, 10, 100	Cui et al[6]
iAs^{III}	WT	0.01, 0.25	Dheer et al[7]
iAs^{III}	WT	18.75, 37.5, 62.5	Garcia-Montalvo et al[8]
iAs^{III}	WT	10	Lu et al[9]
iAs^{III}	WT, IL10-KO	10	Lu et al[10]
iAs^{III}	WT	10	Lu et al[11]
iAs^{III}	CD1	6, 12, 24	Tokar et al[12]
iAs^{III}	CD1	0.05, 0.5, 5	Waalkes et al[13]
iAs^{III}, iAs^{V}	WT, As3mt-KO	25, 100	Dodmane et al[14]
iAs^{V}	WT, As3mt-KO	3.125	Naranmandura et al[15]
iAs^{V}	WT, As3mt-KO	3.125	Drobna et al[16]
iAs^{V}	WT, As3mt-KO	3.125	Hughes et al[17]
iAs^{III}	WT, As3mt-KO	25	Arnold et al[18]
iAs^{III}	WT, As3mt-KO	1, 10, 25, 50	Yokohira et al[19]
iAs^{III}	WT, As3mt-KO	50, 100, 150	Yokohira et al[20]

[a]Exposures reported by Garcia-Montalvo et al, Naranmandura et al, Drobna et al, and Hughes et al were converted from mg As per kg body weight per day to ppm in water based on a 20 gram mouse drinking 3.2 mL per day[8].

Supplementary Table 2. Mantel-Cox comparisons of As3mt-KO survival (100ppm iAs^{V}).

Mantel-Cox Comparison	P value
GF vs. Sham	0.0004
GF vs. Donor A	0.0014
GF vs. Donor B	0.0014
GF vs. Donor C	>0.0001
GF vs. Donor D	>0.0001
GF vs. Donor E	0.0003
Sham vs. Donor A	0.3830
Sham vs. Donor B	0.0189
Sham vs. Donor C	0.3509
Sham vs. Donor D	0.0006
Sham vs. Donor E	0.0040
Donor A vs. B	0.0197
Donor A vs. C	0.1257
Donor A vs. D	0.0030
Donor A vs. E	0.0088
Donor B vs. C	0.0240
Donor B vs. D	0.4382
Donor B vs. E	0.3079
Donor C vs. D	0.0009
Donor C vs. E	0.0024
Donor D vs. E	0.0946

Supplementary Table 3. Survival summary of humanized As3mt-KO groups.

Group	# subjects	# deaths	Median survival (days)
Germ-Free	8	8	5
Sham	9	9	17
Donor A	5	5	17
Donor B	5	5	32
Donor C	14	14	21
Donor D	13	13	30
Donor E	7	7	36

Supplementary Table 4. ANOSIM testing. Between donor-groups (top) and time-point (day 0 vs. day 7) microbiome communities.

Pairwise donor-group comparisons		
Comparison	ANOSIM R	p-value
Global donor effect	0.8314	0.001
Donor A vs. B	1	0.001
Donor A vs. C	0.9916	0.001
Donor A vs. D	0.5571	0.001
Donor A vs. E	0.9348	0.001
Donor B vs. C	0.9941	0.001
Donor B vs. D	0.7776	0.001
Donor B vs. E	0.9354	0.001
Donor C vs. D	0.997	0.001
Donor C vs. E	0.997	0.001
Donor D vs. E	0.0995	0.001
Time-point comparisons (Day 0 vs. Day 7)		
Comparison	ANOSIM R	p-value
Global time-point effect	0.1926	0.001
Donor A	0.536	0.009
Donor B	0.0792	0.008
Donor C	0.5849	0.001
Donor D	0.9807	0.001
Donor E	0.5933	0.002

Supplementary Table 5. Murine modeling details. Sex and age of each co-housed (i.e. cage) mouse group used. Groups represented in multiple figure panes are marked by superscripts described as endnotes to the table.

Figure	Panel	Genotype	Treatment	iAs^V (ppm)	N	Sex	Age (weeks)
1	a	WT	Cef	25	5	Male	10
1	a	WT	Cef	25	5	Female	10
1	a	WT	Sham	25	5	Male	10
1	a	WT	Sham	25	5	Female	10
1	a	WT	Cef	100	5	Female	7
1	a	WT	Sham	100	5	Female	7
1[1]	b	WT	Cef	25	5	Male	10
1[1]	b	WT	Cef	25	5	Female	10
1[1]	b	WT	Sham	25	4	Male	10
1[1]	b	WT	Sham	25	5	Female	10
1	b	WT	Cef	100	3	Male	8
1[2]	b	WT	Cef	100	5	Female	7
1	b	WT	Sham	100	5	Male	8
1[2]	b	WT	Sham	100	5	Female	7
2	a	As3mt	Cef	25	5	Male	13
2	a	As3mt	Cef	25	5	Female	12
2	a	As3mt	Cef	25	5	Male	12
2	a	As3mt	Cef	25	5	Female	13
2	a	As3mt	Sham	25	5	Male	11
2	a	As3mt	Sham	25	5	Female	13
2	a	As3mt	Sham	25	5	Male	12
2	a	As3mt	Sham	25	5	Female	15
2	b	As3mt	Cef	100	5	Male	9
2	b	As3mt	Cef	100	5	Female	11
2	b	As3mt	Sham	100	4	Male	10
2	b	As3mt	Sham	100	5	Female	10
2	c	As3mt	Sham	10	5	Female	11
2	c	As3mt	GF	10	4	Female	11
2	c	As3mt	GF	10	2	Female	10
2	c	As3mt	GF	10	3	Female	10
2	c	As3mt	GF	25	2	Male	7
2	c	As3mt	GF	25	2	Female	7
2	c	As3mt	GF	100	2	Male	12
2	c	As3mt	GF	100	3	Female	12
2	c	As3mt	GF	100	3	Female	17
3[3]	a	As3mt	Sham	100	4	Male	10
3[3]	a	As3mt	Sham	100	5	Female	10
3	a	As3mt	GF-F0	100	5	Male	14
3	a	As3mt	GF-F0	100	4	Female	8
3	a	As3mt	GF-F1	100	2	Male	7
3	a	As3mt	GF-F1	100	5	Female	7
3	a	As3mt	GF-F1	100	5	Female	7
3[3]	a	As3mt	GF	100	2	Male	12
3[3]	a	As3mt	GF	100	3	Female	12
3[3]	a	As3mt	GF	100	3	Female	17
Figure	Panel	Genotype	Treatment	iAs^V (ppm)	No. of Mice	Sex	Age (weeks)

3	b	As3mt	GF-Donor A	100	2	Male	7
3	b	As3mt	GF-Donor A	100	3	Female	7
3	b	As3mt	GF-Donor B	100	2	Male	7
3	b	As3mt	GF-Donor B	100	3	Female	7
3	b	As3mt	GF-Donor C	100	2	Male	8
3	b	As3mt	GF-Donor C	100	5	Female	8
3	b	As3mt	GF-Donor C	100	4	Male	9
3	b	As3mt	GF-Donor C	100	3	Female	9
3	b	As3mt	GF-Donor D	100	4	Male	6
3	b	As3mt	GF-Donor D	100	2	Female	6
3	b	As3mt	GF-Donor D	100	3	Male	9
3	b	As3mt	GF-Donor D	100	4	Female	9
3	b	As3mt	GF-Donor E	100	4	Male	8
3	b	As3mt	GF-Donor E	100	3	Female	8
3[3]	b	As3mt	Sham	100	4	Male	10
3[3]	b	As3mt	Sham	100	5	Female	10
3[3]	b	As3mt	GF	100	2	Male	12
3[3]	b	As3mt	GF	100	3	Female	12
3[3]	b	As3mt	GF	100	3	Female	17
6	a	As3mt	GF-*E. coli*	25	4	Female	17
6	a	As3mt	GF-*F. prausnitzii*	25	3	Male	17
6	a	As3mt	GF-*F. prausnitzii*	25	3	Female	17
6	a	As3mt	GF-*F. prausnitzii*	25	5	Female	9
6	a	As3mt	GF	25	2	Male	7
6	a	As3mt	GF	25	2	Female	7
6	a	As3mt	GF	25	3	Female	15

[1]These mice were used to quantify both fecal and tissue levels of arsenic, with the exception of tissue for one mouse in the Sham-treated group. Tissue samples for this mouse were not collected.
[2]These mice were used to quantify both fecal and tissue levels of arsenic. A separate experiment was conducted using males (3 Cef-treated, 5 Sham-treated) to add data for tissue levels of arsenic.
[3]Survival of groups in figure 2b (i.e. As3mt, Sham-treated, 100 ppm) and 2c (i.e. As3mt, GF, 100 ppm) was included in figures 3a and 3b for comparison.

Supplementary Table 6. PCR primer sets.

Target	Primer	Sequence 5' - 3'	Reference
Universal Bacterial 16S gene	27 F<br>1492 R	AGAGTTTGATCCTGGCTCAG<br>GGTTACCTTGTTACGACTT	Schmidt and Relman
F. prausnitzii (16S)	Fpra428 F<br>Fpra583 R	TGTAAACTCCTGTTGTTGAGGAAGATAA<br>GCGCTCCCTTTACACCCA	Lopez-Siles et al.
Bacterial 16S variable region 4	515 F<br>806R	GTGCCAGCMGCCGCGGTAA<br>GGACTACHVGGGTWTCTAAT	Caporaso et al.

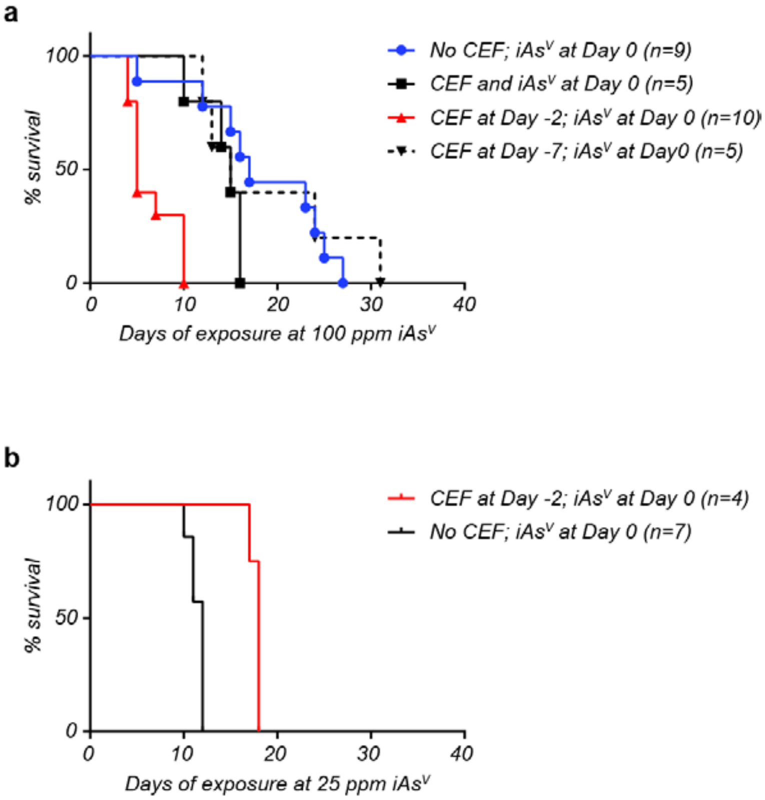

Supplementary Figure 1. The influence of cefoperazone on iAs^V exposure. Combinatorial cefoperazone (Cef, 0.5 mg per mL) and iAs^V (25 ppm) treatment in conventional and germ free (GF) As3mt-KO mice was evaluated in two different experiments. First, survival in conventional As3mt-KO mice during iAs^V exposure was compared between mice pretreated with Cef for 7 (Day -7) or 2 (Day -2) days, mice given Cef on the same day of iAs^V exposure (Day 0), and mice that did not received Cef (**a**). Single or replicate experiments were conducted using four or five mice per experiment as indicated in the legend. Survival in the Day -2 Cef treatment group was significantly shorter compared to mice that did not receive Cef (p=0.0002, Mantel-Cox test), whereas survival in the other Cef treatment groups was not significantly different (Day 7, p=0.7192; Day 0, p=0.0894; Mantel-Cox test). These results in conventional mice suggest that the influence of Cef is greatest when administered 2 days prior to iAs^V exposure at the point in which the microbiome is significantly altered. In a second experiment (**b**), survival in GF As3mt-KO mice (one cage of four mice) was compared between mice that received the Day -2 Cef treatment and mice that did not receive Cef (two cages of three or four mice). Survival was significantly shorter in mice that did not receive Cef (p=0.0043, Mantel-Cox test), suggesting that Cef pretreatment did not significantly increase toxicity. Collectively, these results provide evidence that there was minimal additive synergistic effects on toxicity when Cef and iAs^V were co-administered in this murine model.

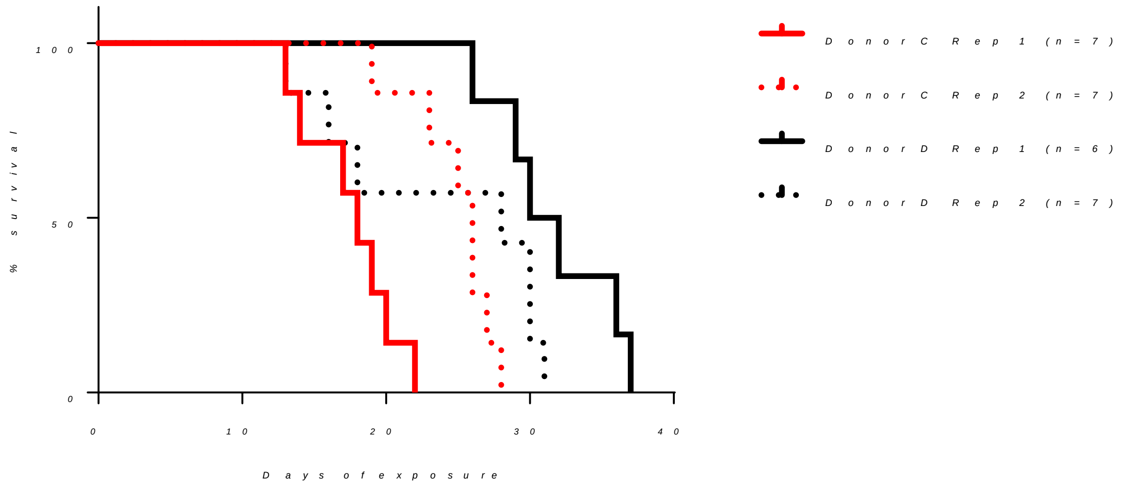

Supplementary Figure 2. Replicate exposures of As3mt-KO humanized donor groups. In the first experiment (Rep 1), median survival of As3mt-KO mice receiving stool transplantation from donor D was significantly longer following arsenic exposure (iAs^V, 100 ppm) compared to mice receiving stool transplantation from donor C (p=0.0004, Mantel-Cox test). Repeating the experiment (Rep 2) and doubling the overall number of mice in the comparison (aggregate data) did not change this result (p=0.0009, Mantel-Cox test), even though the median survival of group C mice was significantly longer in the second experiment (p=0.0009, Rep 1 vs. Rep 2, Mantel-Cox test), and was not significantly different from group D mice in the second experiment (p=0.2268, Group C Rep 2 vs. Group D Rep 2, Mantel-Cox test). All p-values (Mantel-Cox test) are given below.

	Group C, Rep 1	*Group C, Rep 2*	*Group D, Rep 1*	*Group D, Rep 2*
Group C, Rep 1				
Group C, Rep 2	0.0009			
Group D, Rep 1	0.0004	0.0027		
Group D, Rep 2	0.0736	0.2268	0.0603	

Aggregate Group C vs. Aggregate Group D: p=0.0009 (Aggregate = all mice from both experiments)

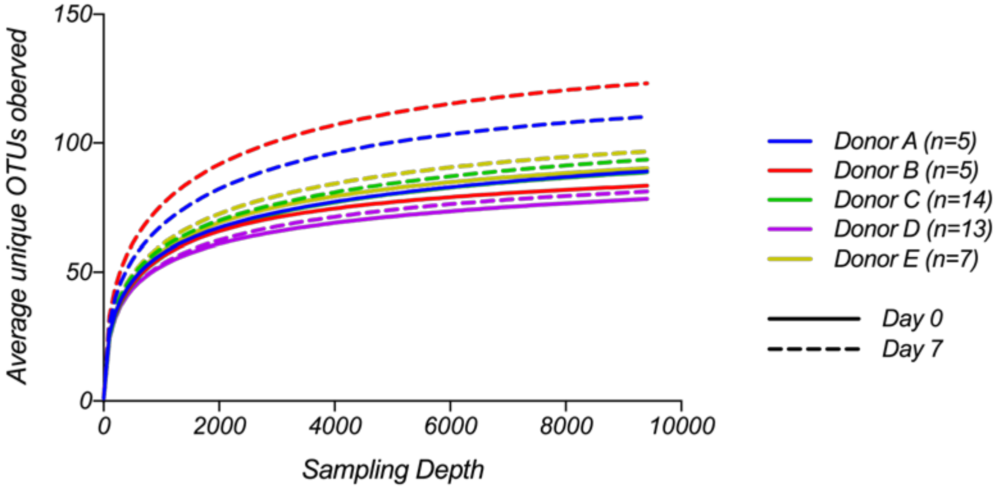

Supplementary Figure 3. Rarefaction of OTUs from 16S rRNA (V4) sequence data. Rarefaction analysis of the fecal microbiome of humanized mice on days 0 (solid lines) and 7 (hashed lines) generated by random subsampling to a depth of 9408. Adequate coverage was obtained for each sample (>99% Good's coverage estimate). Observed species richness ranges from 64 to 128, with ACE richness estimates between 66 and 128.

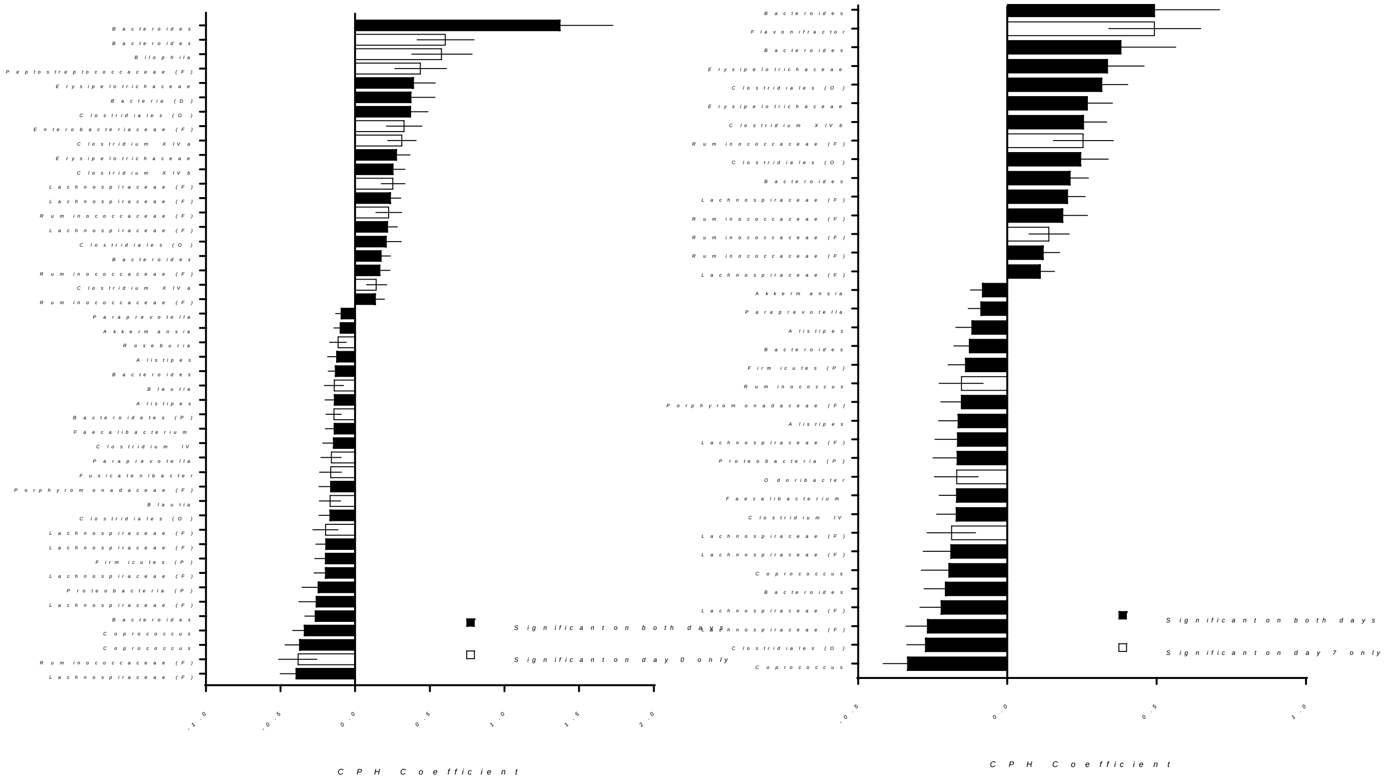

Supplementary Figure 4. Survival modeling against OTU abundance. Log-transformed abundance of the 100 most abundant OTUs were fit to univariate survival models (Cox proportional hazards). Controlling for false discovery rate (<0.1), significant correlations between OTU abundance and survival are represented here. Bars represent the correlation (beta) coefficient estimate with standard error represented. Correlations identified on both day 0 and day 7 are presented. Black bars were correlated at both time points. Negative coefficients are associated with increased survival (decreased hazard).

a

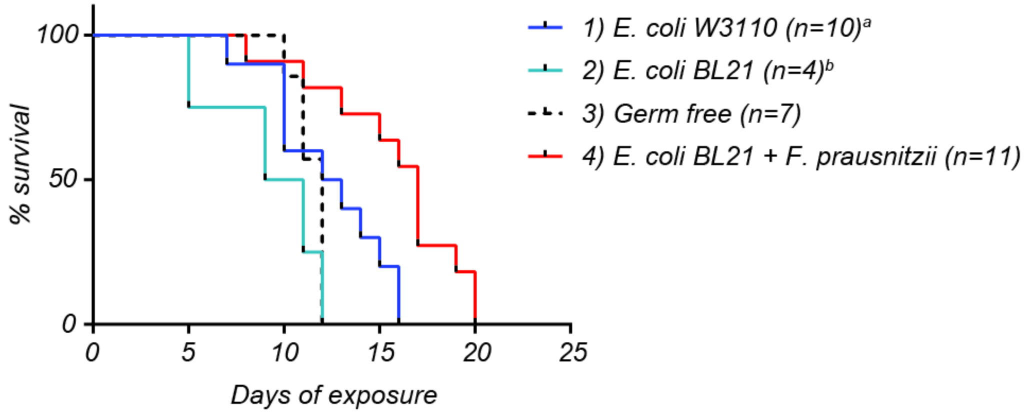

b

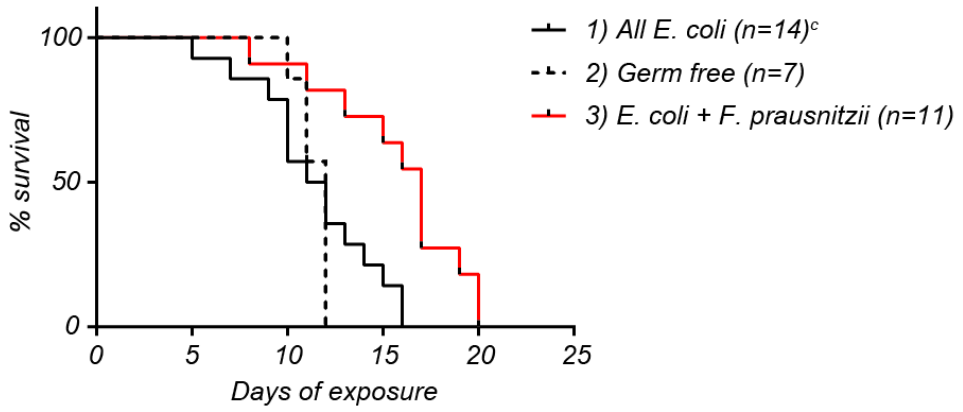

Supplementary Figure 5. Survival in groups of GF, *E. coli* mono-colonized, *E. coli* + *F. prausnitzii* bi-colonized mice. Survival of mice mono-colonized with two different *E. coli* strains (W3110 and BL21) are shown separately in **a**, or as pooled data in **b**. Survival of both *E. coli* mono-colonized groups of mice in **a** was not significantly different from GF mice (p=0.2102, W3110; p=0.1759, BL21; Mantel-Cox test). Survival of pooled *E. coli* mono-colonized mice in **b** was not different from GF mice (p=0.5791, Mantel-Cox test). Survival of *E. coli* + *F. prausnitzii* bi-colonized mice was longer compared to GF mice (p=0.0035, same groups shown in **a** and **b**; Mantel-Cox test) and *E. coli* mono-colonized mice (p=0.0080, W3110 in **a**; p=0.0024, BL21 in **a**; p=0.0018, all *E. coli* in **b**; Mantel-Cox test). Results reported in the main body of the manuscript (Figure 4 panel e) represent data from *E. coli* BL21 only. [a]W3110 carried the empty cloning vector, pMal-c5x, and was maintained in mice with ampicillin (100 µg per mL) in drinking water. [b]BL21 carried the empty cloning vector, pET29a, and was maintained in mice with kanamycin (50 µg per mL) in drinking water. [c]All *E. coli* refers to pooled W3110 and BL21 data from panel a.

Supplementary References

1 Kenyon, E. M. *et al.* How can biologically-based modeling of arsenic kinetics and dynamics inform the risk assessment process? - A workshop review. *Toxicology and applied pharmacology* **232**, 359-368, doi:10.1016/j.taap.2008.06.023 (2008).

2 ATSDR. (ed Health and Human Services) (Atlanta, GA, 2007).

3 Center for Drug Evaluation and Research, C. f. B. E. a. R. (ed US Food and Drug Administration) (Rockville, MD, USA, 2002).

4 Freireich, E. J., Gehan, E. A., Rall, D. P., Schmidt, L. H. & Skipper, H. E. Quantitative comparison of toxicity of anticancer agents in mouse, rat, hamster, dog, monkey, and man. *Cancer chemotherapy reports* **50**, 219-244 (1966).

5 Apata, M., Arriaza, B., Llop, E. & Moraga, M. Human adaptation to arsenic in Andean populations of the Atacama Desert. *American journal of physical anthropology* **163**, 192-199, doi:10.1002/ajpa.23193 (2017).

6 Cui, X., Wakai, T., Shirai, Y., Hatakeyama, K. & Hirano, S. Chronic oral exposure to inorganic arsenate interferes with methylation status of p16INK4a and RASSF1A and induces lung cancer in A/J mice. *Toxicological sciences : an official journal of the Society of Toxicology* **91**, 372-381, doi:10.1093/toxsci/kfj159 (2006).

7 Dheer, R. *et al.* Arsenic induces structural and compositional colonic microbiome change and promotes host nitrogen and amino acid metabolism. *Toxicology and applied pharmacology* **289**, 397-408, doi:10.1016/j.taap.2015.10.020 (2015).

8 Garcia-Montalvo, E. A., Valenzuela, O. L., Sanchez-Pena, L. C., Albores, A. & Del Razo, L. M. Dose-dependent urinary phenotype of inorganic arsenic methylation in mice with a focus on trivalent methylated metabolites. *Toxicology mechanisms and methods* **21**, 649-655, doi:10.3109/15376516.2011.603765 (2011).

9 Lu, K. *et al.* Gut microbiome perturbations induced by bacterial infection affect arsenic biotransformation. *Chemical research in toxicology*, doi:10.1021/tx4002868 (2013).

10 Lu, K. *et al.* Gut microbiome phenotypes driven by host genetics affect arsenic metabolism. *Chemical research in toxicology* **27**, 172-174, doi:10.1021/tx400454z (2014).

11 Lu, K. *et al.* Arsenic exposure perturbs the gut microbiome and its metabolic profile in mice: an integrated metagenomics and metabolomics analysis. *Environmental health perspectives* **122**, 284-291, doi:10.1289/ehp.1307429 (2014).

12 Tokar, E. J., Diwan, B. A., Ward, J. M., Delker, D. A. & Waalkes, M. P. Carcinogenic effects of "whole-life" exposure to inorganic arsenic in CD1 mice. *Toxicological sciences : an official journal of the Society of Toxicology* **119**, 73-83, doi:10.1093/toxsci/kfq315 (2011).

13 Waalkes, M. P., Qu, W., Tokar, E. J., Kissling, G. E. & Dixon, D. Lung tumors in mice induced by "whole-life" inorganic arsenic exposure at human-relevant doses. *Archives of toxicology* **88**, 1619-1629, doi:10.1007/s00204-014-1305-8 (2014).

14 Dodmane, P. R. *et al.* Characterization of intracellular inclusions in the urothelium of mice exposed to inorganic arsenic. *Toxicological sciences : an official journal of the Society of Toxicology* **137**, 36-46, doi:10.1093/toxsci/kft227 (2014).

15 Naranmandura, H., Rehman, K., Le, X. C. & Thomas, D. J. Formation of methylated oxyarsenicals and thioarsenicals in wild-type and arsenic (+3 oxidation state) methyltransferase knockout mice exposed to arsenate. *Analytical and bioanalytical chemistry* **405**, 1885-1891, doi:10.1007/s00216-012-6207-0 (2013).

16 Drobna, Z. *et al.* Disruption of the arsenic (+3 oxidation state) methyltransferase gene in the mouse alters the phenotype for methylation of arsenic and affects distribution and retention of orally administered arsenate. *Chemical research in toxicology* **22**, 1713-1720, doi:10.1021/tx900179r (2009).

17 Hughes, M. F. *et al.* Arsenic (+3 oxidation state) methyltransferase genotype affects steady-state distribution and clearance of arsenic in arsenate-treated mice. *Toxicology and applied pharmacology* **249**, 217-223, doi:10.1016/j.taap.2010.09.017 (2010).
18 Arnold, L. L. *et al.* Time Course of Urothelial Changes in Rats and Mice Orally Administered Arsenite. *Toxicologic pathology*, doi:10.1177/0192623313489778 (2013).
19 Yokohira, M. *et al.* Effect of sodium arsenite dose administered in the drinking water on the urinary bladder epithelium of female arsenic (+3 oxidation state) methyltransferase knockout mice. *Toxicological sciences : an official journal of the Society of Toxicology* **121**, 257-266, doi:10.1093/toxsci/kfr051 (2011).
20 Yokohira, M. *et al.* Severe systemic toxicity and urinary bladder cytotoxicity and regenerative hyperplasia induced by arsenite in arsenic (+3 oxidation state) methyltransferase knockout mice. A preliminary report. *Toxicology and applied pharmacology* **246**, 1-7, doi:10.1016/j.taap.2010.04.013 (2010).
